# Ultra-Wideband High-Efficiency Solar Absorber and Thermal Emitter Based on Semiconductor InAs Microstructures

**DOI:** 10.3390/mi14081597

**Published:** 2023-08-14

**Authors:** Yanying Zhu, Pinggen Cai, Wenlong Zhang, Tongyu Meng, Yongjian Tang, Zao Yi, Kaihua Wei, Gongfa Li, Bin Tang, Yougen Yi

**Affiliations:** 1 Joint Laboratory for Extreme Conditions Matter Properties, Tianfu Institute of Research and Innovation, State Key Laboratory of Environmental Friendly Energy Materials, Key Laboratory of Manufacturing Process Testing Technology of Ministry of Education, Southwest University of Science and Technology, Mianyang 621010, China; 13605854753@163.com (Y.Z.); zwl2964875412@163.com (W.Z.); tangyongjian2000@sina.com (Y.T.); 2 Department of Applied Physics, College of Science, Zhejiang University of Technology, Hangzhou 310023, China; caippgg@zjut.edu.cn; 3 Leicester International Institute, Dalian University of Technology, Dalian 124221, China; 18245918766@163.com; 4 School of Chemistry and Chemical Engineering, Jishou University, Jishou 416000, China; 5 School of Automation, Hangzhou Dianzi University, Hangzhou 310018, China; 6 Key Laboratory of Metallurgical Equipment and Control Technology of Ministry of Education, Wuhan University of Science and Technology, Wuhan 430081, China; ligongfa@wust.edu.cn; 7 School of Microelectronics and Control Engineering, Changzhou University, Changzhou 213164, China; btang@cczu.edu.cn; 8 College of Physics and Electronics, Central South University, Changsha 410083, China; yougenyi@csu.edu.cn

**Keywords:** solar absorber, metamaterial, ultra-wideband absorption, thermal emitter, polarization insensitivity, semiconductor

## Abstract

Since the use of chemical fuels is permanently damaging the environment, the need for new energy sources is urgent for mankind. Given that solar energy is a clean and sustainable energy source, this study investigates and proposes a six-layer composite ultra-wideband high-efficiency solar absorber with an annular microstructure. It achieves this by using a combination of the properties of metamaterials and the quantum confinement effects of semiconductor materials. The substrate is W–Ti–Al_2_O_3_, and the microstructure is an annular InAs-square InAs film–Ti film combination. We used Lumerical Solutions’ FDTD solution program to simulate the absorber and calculate the model’s absorption, field distribution, and thermal radiation efficiency (when it is used as a thermal emitter), and further explored the physical mechanism of the model’s ultra-broadband absorption. Our model has an average absorption of 95.80% in the 283–3615 nm band, 95.66% in the 280–4000 nm band, and a weighted average absorption efficiency of 95.78% under AM1.5 illumination. Meanwhile, the reflectance of the model in the 5586–20,000 nm band is all higher than 80%, with an average reflectance of 94.52%, which has a good thermal infrared suppression performance. It is 95.42% under thermal radiation at 1000 K. It has outstanding performance when employed as a thermal emitter as well. Additionally, simulation results show that the absorber has good polarization and incidence angle insensitivity. The model may be applied to photodetection, thermophotovoltaics, bio-detection, imaging, thermal ion emission, and solar water evaporation for water purification.

## 1. Introduction

Although economic globalization has accelerated since the United Nations Conference on Environment and Development in Rio de Janeiro in 1992 [1], the challenges facing humanity over the topic of sustainable development have gotten more serious [2]. People require more energy supply when industrialized society develops so quickly. However, the use of chemical fuels like coal and oil, which are primarily used for energy supply, has undoubtedly significantly increased the amount of carbon dioxide in the air, causing global warming [3], disrupting the ecological environment system’s balance, resulting in the deterioration of the ecological environment, and increasing the frequency of natural disasters. For instance, recent years have seen a worsening of global climate change, resulting in regular calamities like floods and droughts [4]. It is critical to develop new forms of energy. Solar energy, which is a green and clean energy source, is unquestionably the leading contender in the battle for new energy development. The study of more effective solar energy utilization has attracted a lot of attention [5,6,7].

How efficiently solar energy is absorbed is the key to effective solar energy utilization. The most well-known solar energy absorption tool is solar absorption coating. It works by using the absorption function of special materials for solar energy. The main problem with this type of coated solar absorber is that it is not resistant to high temperatures. High temperature resistant absorber coatings are also a hot research topic in the field of solar absorber coatings. In 2023, Patel et al. designed a multi-layer Ti Si solar absorber that can be used for solar thermal energy conversion [8]. Another class of solar absorbers is the plasma solar absorber based on nanostructured materials. This absorber is mainly used to make materials that are not good absorbers of solar energy into good absorbers of solar energy by changing the surface structure of the material, such as black silicon and black diamond with double nano-texture. There are many processes to process such materials, for example, in 2019, Chen et al. used chemical etching combined with vapor phase degalvanization to fabricate porous Ni solar absorbers [9]. In 2023, Patel et al. built a Graphene based corundum shaped solar absorber that can be used in the UV to MIR region [10]. In 2022, Chen et al. used reactive ion etching to fabricate black germanium solar absorbers [11]. In 2020, Mastellone et al. used femtosecond pulsed laser processing to fabricate single-crystal 6H–SiC solar absorber [12]. The disadvantage of this type of absorber is also that it is not resistant to high temperatures. Under high temperature operating conditions, the modified material surface can easily undergo phase transformation to destroy the nanostructure and thus lose the absorption performance. Metamaterial broadband solar absorber is a hot research topic in the field of solar absorber in recent years. It does not achieve the modification by etching the surface of the material. Instead, it chooses to stack subwavelength microstructures with specific geometries on the material surface for the purpose of solar energy absorption. Metamaterials are artificially designed subwavelength composites that possess extraordinary physical properties different from those of natural materials and whose properties are mainly determined by the artificially designed microstructure [13,14]. Researchers have used the special properties of metamaterials to achieve special functions such as ultrasonic focusing [15] or to create special materials such as heat dissipation materials [16] and invisibility cloaks [17]. In 2008, the concept of metamaterial perfect absorber was first introduced by Landy et al. [18]. This absorber consists of a metallic resonant ring and a cut line with a single frequency absorption peak. Since then, research related to metamaterial-based solar absorbers has mushroomed [19,20,21]. For example, in 2022, Chen et al. reported a broadband solar absorber with a four-layer structure [22]. In 2021, Huang et al. designed an ultra-wideband solar absorber in 500–2850 nm band with a GaAs grating composite structure based on W–Ti–GaAs [23]. In 2022, Wang et al. designed a Ni material crossover microstructure for a solar broadband absorber [24].

The microstructure of metamaterial solar absorbers is the key to achieve efficient absorption. By adjusting the microstructure, the absorber’s absorption performance can be adjusted for different wavelength bands. Therefore, metamaterial absorbers have more flexibility compared to conventional solar absorbers. However, the theory related to the metamaterial absorber is not perfect at present. Therefore, the design of the metamaterial absorber is somewhat contingent. When a model is designed that does not have a satisfactory absorption efficiency in a certain band, researchers generally choose to find a more suitable structure through multiple trials. This process is generally very time and energy consuming. Therefore, we propose a semiconductor top microstructure-metal-insulator-metal structure (SMIM) for a metamaterial broadband solar absorber. The advantage of this structure is that the structural parameters of the top semiconductor microstructure can be adjusted to improve the absorption efficiency in a certain band in a directional manner. The principle used is that the quantum-limited [25] effect of nanosemiconductor materials causes a blue shift in the absorption band of the material and thus increases the absorption efficiency in a specific band [26,27]. When the size of a nanosemiconductor material is smaller than the exciton Bohr diameter $\alpha_{B}$, the material shows quantum-limited effects. Different semiconductor materials have different exciton Bohr diameters. The tuning of the model can be achieved in a time-saving way by selecting the right semiconductor material. This can also provide a new idea for the optimization of metamaterial broadband solar absorbers.

Meanwhile, according to Kirchhoff’s law of thermal radiation, the thermal emissivity of any object is equal to its light absorption rate. After calculation, the thermal radiation spectrum of our model in the 280–2750 nm band is almost identical to the ideal blackbody radiation spectrum. This indicates that the model has a good photothermal conversion efficiency and is well suited as a heat emitter matching the solar spectrum [28,29]. This has important implications for the field of thermophotovoltaic power generation. In addition, the model has a high reflectivity in the thermal infrared band. This allows the model to work stably even under high temperature radiation operating conditions. The selectivity of the spectra makes the models have a higher potential for applications compared to conventional solar absorbers.

In this paper, a six-layer composite ultra-broadband high-efficiency solar absorber based on a semiconductor multilayer ring structure is proposed. W–Ti–Al_2_O_3_ is used as the substrate, and the annular InAs-square InAs film-Ti film is used as the microstructure. Considering the practical performance of the product, the model chooses W as the substrate, which has low transmittance and can greatly reduce the transmitted light and increase the light absorption. And the melting points of W, metal Ti and insulator Al_2_O_3_ are 3410 °C, 1668 °C and 2054 °C, respectively [30], which have good fire resistance and stable chemical properties, so the service life of the model is also longer. In addition, the larger the exciton Bohr diameter, the easier it is to produce quantum-limited effects, while the exciton Bohr diameter of group III–V semiconductors is generally larger [31]. At the same time, to ensure that the semiconductor can effectively excite excitons in sunlight, we should choose a semiconductor with a narrow band gap. So after reviewing the relevant information, we chose the group III–V semiconductor InAs, which has an exciton Bohr diameter of 62–74 nm and a band gap of 0.36 eV [32]. We simulated the model using FDTD software from Lumerical Solutions. The obtained results show that the absorption of the model in the 283–3615 nm band all exceeds 90%, with an absorption bandwidth of 3332 nm and an average absorption of 95.80%, and an average absorption of 95.66% in the whole radiation band of the sunlight from 280–4000 nm. The weighted average absorption rate under AM1.5 light was 95.78%. Meanwhile, the reflectance of the model in the 5586–20,000 nm band is all higher than 80%, with an average reflectivity of 94.52%. This indicates that the model has good spectral selectivity and excellent thermal infrared suppression ability. Since the microstructure of the model is a highly symmetric annular structure, the model has polarization insensitivity. We also simulate the absorption properties of the model when the incident light is incident at 0–60°. The results show that the model has good wide-angle absorption properties. Compared with most solar absorbers, our proposed solar absorber has a wider absorption bandwidth with good thermal emission properties, which broadens its application areas with high potential not only for imaging and photodetection, but also for thermal emission, thermophotovoltaics, biomedical detection and solar water evaporation and purification.

## 2. Model Designing and Digital Modeling

**Figure 1:**
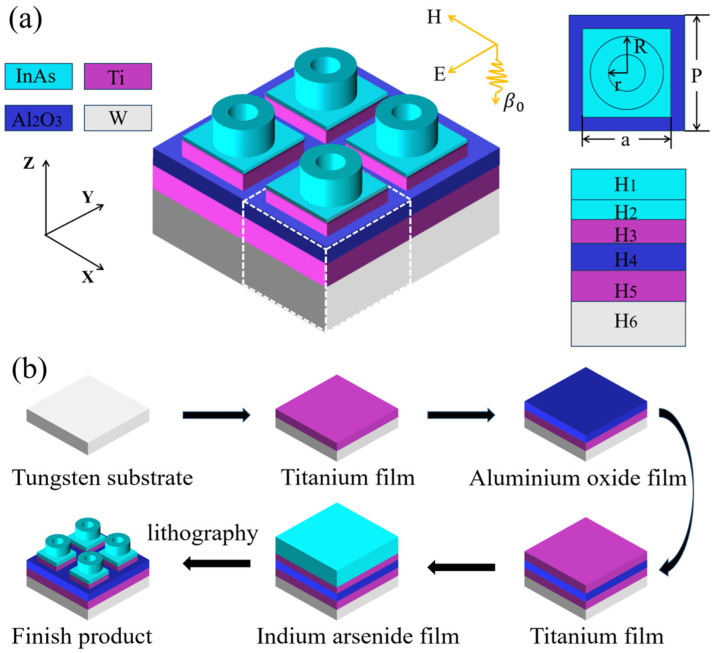
(**a**) 3D model and model section in XOY direction. (H_1_ = 190 nm, H_2_ = 50 nm, H_3_ = 80 nm, H_4_ = 120 nm, H_5_ = 160 nm, H_6_ = 400 nm, P = 400 nm, a = 300 nm, R = 120 nm, r = 83 nm). (**b**) This is the design of the preparation process of the model.

The construction is illustrated in Figure 1a which is the nanostructure proposed, with W–Ti–Al_2_O_3_ as the substrate and annular InAs-square InAs film–Ti film as the microstructure. The relative permittivity of all materials used in this paper was taken from the Palik manual [33]. When FDTD is used for simulation calculation, we choose plane wave as the light source, and periodic boundary conditions as the boundary conditions in X and Y directions, the grid accuracy is designed as 5 rad. P, the structure period is 400 nm, r, the inner diameter of the annular is 83 nm, R, the outer diameter of the annular is 120 nm, a, the side length of the square is 300 nm, the thickness H_1_ = 190 nm, H_2_ = 50 nm, H_3_ = 80 nm, H_4_ = 120 nm, H_5_ = 160 nm, H_6_ = 400 nm. Here the 400 nm thick W material is mainly used to reduce transmitted light to increase light absorption. When the thickness of W is sufficient, the transmittance T approaches zero, and the absorption A = 1–T–R (T is the transmittance and R is the reflectivity) [34,35,36,37]. When T approaches zero, it is possible to calculate the absorption A using A = 1 − R. In the selection of the inner and outer diameter of the annular InAs, we have to ensure that the difference between the inner diameter (r) and the outer diameter (R), and the thickness of the square InAs film (H_2_) is less than the exciton Bohr diameter of the InAs material (62–74 nm) so that the semiconductor quantum confinement effect can occur.

At present, this study is only at the stage of theoretical modeling. Considering the in-depth study and practical production of the model at a later stage, we propose to make a theoretically feasible process of making the finished model. Figure 1b is the production flow of the model. In the preliminary stage, we use RCA cleaning method to clean the substrate with 1 # liquid (NH_4_OH:H_2_O_2_:H_2_O = 1:2:6) and 2 # liquid (HCl:H_2_O_2_:H_2_O = 1:2:6), then rinse with deionized water, and finally dry [38]. After the preparatory work is completed, A 170 nm thick Ti film was deposited by magnetron sputtering on a 400 nm thick W substrate, and then an Al_2_O_3_ film with a thickness of 120 nm, a Ti film with a thickness of 80 nm, and an InAs film with a thickness of 130 nm are sequentially deposited on the surface of the Ti film by ion beam sputtering. Next, the desired microstructure is obtained by Electron Beam Lithography (pre baking and primer coating, photoresist coating, pre baking, alignment, exposure, post baking, development, hard film, pattern detection). Finally, ion beam etching was used to obtain the solar absorber with W–Ti–Al_2_O_3_ as the substrate and annular InAs-square InAs film–Ti film as the microstructure [39]. It is worth mentioning that the development of metamaterials cannot be separated from the progress of the fine processing industry. And lithography is the key in the field of fine processing. Currently it is not easy to process structures within 100 nm. Electron beam lithography is one of the feasible solutions. Since the wavelength of the electron beam under the high-pressure acceleration system is up to 0.14 nm, the shorter the working wavelength according to Rayleigh criterion is the basis for obtaining high resolution. This undoubtedly increases the technical difficulty of the process, and the time cost, so this technology is currently not suitable for mass production. As a result, most metamaterial products are still in the laboratory stage. However, with the development of fine processing technology, the application prospect of metamaterial products in the future is quite bright.

## 3. Conclusions Analysis and Discussion

**Figure 2:**
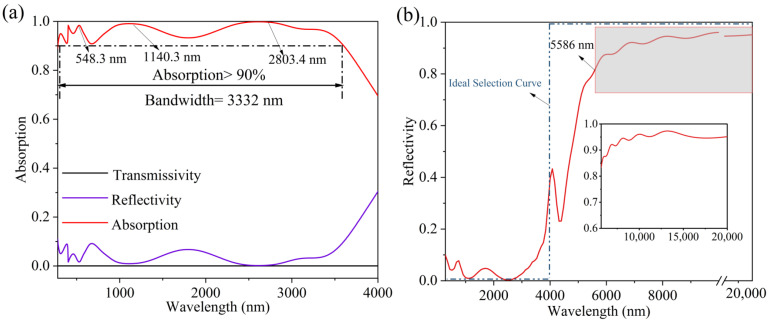
(**a**) Transmissivity, reflectivity and absorption spectrum comparison chart. (**b**) The reflectivity of the model in the 280–2000 nm wavelength range.

After finishing the modeling in FDTD, we select the planar light of 280–4000 nm for simulation. Figure 2a shows the calculated results. From the figure, we can see that the transmittance does tend to zero. At the same time, the absorption of the model in the 283–3615 nm band are over 90%, and the absorption bandwidth reaches 3332 nm. The average absorption in the 283–3615 nm band was calculated to be 95.66%. Figure 2b shows the reflectivity map of the model in the 280–20,000 nm band. The reflectance of the models in the band after 5586 nm is all higher than 80%, with an average reflectance of 94.52%. This indicates that the model has good spectral selectivity and excellent thermal infrared suppression. This advantage allows the model to work more stably under high temperature radiation conditions.

We also simulated the absorption spectra of the model under AM1.5 illumination considering the realistic light conditions. The global spectral equations under AM1.5 illumination are as follows [40,41]:

(1) $$\eta_{A}=\frac{\int_{\lambda_{Min}}^{\lambda_{Max}}A(\omega )I_{AM1.5}(\omega )d\omega }{\int_{\lambda_{Min}}^{\lambda_{Max}}I_{AM1.5}(\omega )d\omega }$$

**Figure 3:**
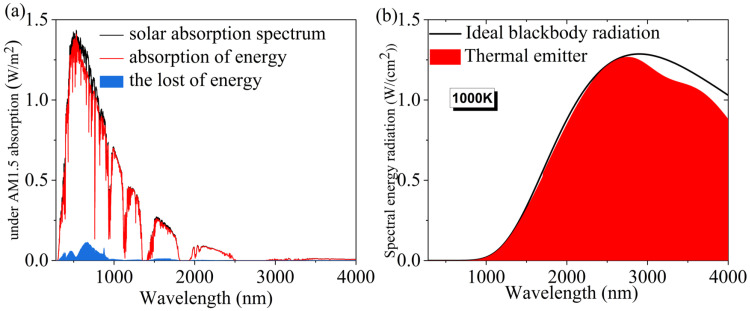
(**a**) Solar energy spectrum under AM1.5 light, absorbed energy spectrum and loss distribution of solar absorber under AM1.5 light. (**b**) Energy emission spectrum of the solar absorber at 1000 K.

The simulation results are shown in Figure 3. As shown in Figure 3a, under AM1.5 illumination, the solar spectrum is shown in black, the absorbed energy spectrum of the model is shown in red, and the lost energy spectrum is shown in blue. After calculation, it can be seen that the weighted average absorption of the model in the 280–4000 nm band is 95.78%. The energy loss is less than 5%. Also, the application of the model in thermophotovoltaic power generation systems is considered. We also simulated the thermal emission efficiency of the model at 1000 K to judge how well its radiation spectrum matches the ideal blackbody radiation spectrum. The simulation results are shown in Figure 3b. From Figure 3b, it can be seen that the thermal radiation spectrum of the model in the wavelength band less than 2750 nm is almost identical to the ideal blackbody radiation spectrum. This indicates that the model has a very good thermal radiation efficiency in this band. And the model’s thermal radiation spectrum in the 2500–4000 nm band matches the ideal blackbody radiation spectrum somewhat less well [42]. This indicates that the energy loss of the model in this band is relatively large. However, since the radiant energy of sunlight in this band is inherently small, the final loss of solar energy is still small for a large energy loss ratio. After calculation, the model has a high thermal radiation efficiency of 95.42% in the 280–4000 nm band. The excellent thermal radiation performance makes the model have a high potential for application in the field of thermophotovoltaic power generation. To show the advantages of this model more clearly, we also compared it with several models of the same type, as shown in Table 1, and it can be seen that the absorption bandwidth of this model is very wide and superior to other models, and the average absorption rate of the model is also very high with such a wide and high absorption bandwidth [43,44,45,46]. It can be said that the prospect of its practical application is very good.

The microstructure of the model is the key for the model to achieve broadband absorption. Under the action of light waves, surface plasmons (SPs) on the surface of the microstructure interact with photons to produce surface plasmonic polaritons (SPPs) [46,47]. SPPs are mixed excited states of free electrons and photons, which contain the electron motion at the material surface and electromagnetic waves in the medium [48]. So SPPs will appear both inside and outside of the material. And the SPs of the material have an inherent vibration frequency with the following equation [49]:

(2) $$\omega_{\rho }=\sqrt{\frac{ne^{2}}{m\varepsilon_{0}}}$$

**Figure 4:**
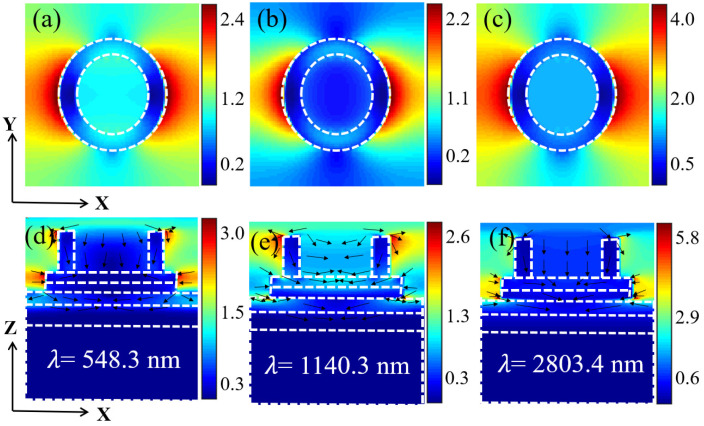
(**a**–**c**) Electric field diagram matching the last three absorption peaks on the XOY plane of solar absorber, (**d**–**f**) electric field diagram matching the last three absorption peaks on the XOZ plane of the solar absorber.

The *n* is the free electron number density. The $\varepsilon_{0}$ is the vacuum dielectric constant. The equipartition exciton resonance occurs when the frequency of the incident light wave is equal to $\omega_{\rho }$, when the absorption of the light wave is the strongest. The plasma resonance can be broadly divided into surface plasma resonances (SPRs) [50] and local surface plasma resonances (LSPRs) [51]. SPRs arise at the metal-insulator interface and will propagate along the interface. Therefore, there is local field enhancement both inside the insulator and at the interface boundary. In contrast, LSPRs will be highly concentrated on the nanoparticle surface, and therefore will appear as a highly concentrated localized field on the nanoparticle surface [52]. The difference between these two resonance absorption modes is also our criterion to distinguish them. Based on the above rationale, the electric field distribution maps corresponding to the three resonances absorption peak wavelengths (548.3 nm, 1140.3 nm, and 2803.4 nm) were selected for analysis to further understand the physical mechanism of broadband absorption in this model. From Figure 4a,d, it can be seen that at the resonance wavelength of 548.3 nm, the enhancement of the local field appears at the outer edge of the upper surface of the annular InAs layer and the second InAs thin layer at the top of the model. This is due to the strong coupling interaction between the light wave and the excitons at the outer edge of the semiconductor, where exciton-photon resonant absorption occurs and local field enhancement of the electric field is realized. As can be seen in Figure 4b,e, the top InAs material still shows strong exciton resonance absorption of electromagnetic waves at the resonance wavelength of 1140.3 nm. The electric field enhancement appears at the interface between the third layer of Ti material and the fourth layer of Al_2_O_3_ material, and there is also a strong electric field distribution inside the Al_2_O_3_ material. This is the absorption enhancement due to the absorption of SPRs. In addition, a stronger electric field distribution also appears inside the second layer of InAs material, but there is no appearance of a local enhancement field in the air, which is known to be neither SPR nor LSPR absorption. Here is where the quantum-limited effect of the nanosemiconductor material comes into play. The absorption wavelength range of normal nanomaterials should satisfy the following equation [53]:

(3) $$\lambda <\frac{hc}{E_{g}}$$

$E_{g}$ is the band gap of the material and *c* is the speed of light. After calculation, the maximum absorption wavelength of the InAs material is 2755.6 nm. Since the thickness of the second layer of InAs material is smaller than its Bohr exciton diameter, its quantum-limited effect leads to a blue-shift in the absorption wavelength, resulting in strong absorption in the near-infrared band [54]. From Figure 4c,f, at the resonance wavelength of 2803.4 nm, it is the exciton resonance absorption occurring at the upper edge of the second layer of InAs material and the SPRs absorption occurring at the interface between the third layer of Ti material and the fourth layer of Al_2_O_3_ material that play a major role. From this, it is clear that SPRs and exciton resonance absorption in nano semiconductor materials corresponding to different wavelengths enhance the coupling of absorption with quantum-limited effects of nanosemiconductor materials, achieving the goal of model broadband efficient absorption. At the same time, the absorption blueshift phenomenon of quantum-limited effect of nanosemiconductor materials can be used to optimize the absorption efficiency of the model in a certain waveband. This also provides a new idea for how to further optimize the absorption efficiency of metamaterial broadband absorbers [55].

**Figure 5:**
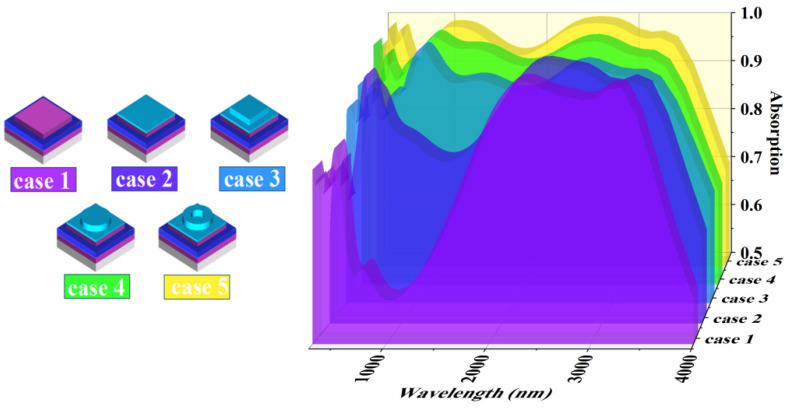
Five solar absorbers with different structures and comparison of absorption of five solar absorbers.

To investigate the superiority of this solar absorber structure, five different structures were studied comparatively, as shown in Figure 5 (Case 1, no semiconductor microstructure. Case 2, no top microstructure. Case 3, square microstructure. Case 4, circular microstructure. Case 5, annular microstructure). After the simulation, the comparison graph of the absorption of the five structures was obtained, as shown in Figure 5. As can be seen from the figure, in Case 1 without semiconductor microstructure, the model has a very low absorption in the wavelength band less than 2000 nm. This further suggests that the quantum-limited effect of the InAs nanosemiconductor material improves the absorption performance of the model in this band. This is also in good agreement with the conclusions of the above analysis regarding the physical mechanism. After adding a layer of InAs film (Case 2) on top of the model in Case 1, the absorption of the model in the 280–2000 nm band was improved, but the absorption performance was still not satisfactory. A layer of square InAs material microstructure is added on top of the model of Case 2 again (Case 3). We can see from Figure 5 that although the model for case 3 improves the absorption in the band around 2000 nm, the absorption in the band where the model wavelength is less than 1000 nm is still unsatisfactory. We tried to replace the square InAs material microstructure with a circular microstructure (Case 4). The model of Case 4 shows better absorption performance in the wavelength band less than 1000 nm. This indicates that a suitable geometry can indeed improve the microstructure’s ability to modulate light waves. Finally, we simulated the absorption performance of the circular InAs material microstructure (Case 5). The absorption performance is better than that of the circular microstructure. This suggests that the model with the annular microstructure is indeed superior to the models for several other cases. In addition, the absorption of the square microstructured absorber is low in the UV and visible wavelengths. Meanwhile, although the absorption of circular microstructure absorbers is better than that of square microstructure absorbers in this band, it is also significantly lower than that of annular microstructure absorbers [56]. In addition, we also calculated the weighted average absorbance of the three models of Cases 1, 2, 3 under AM1.5 light. The results showed that the absorption rate was 91.12% for the model with square microstructure, 94.15% for the model with circular microstructure, and 95.78% for the model with annular microstructure. Thus, the absorption performance of the model with the annular microstructure is indeed the best among the five models in the entire 280–4000 nm band.

**Figure 6:**
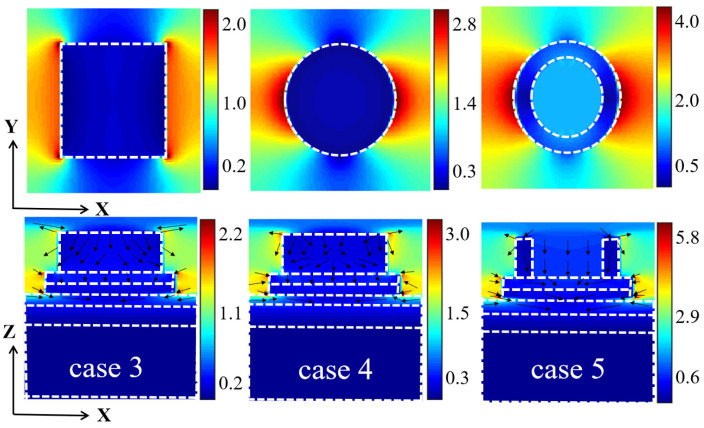
Electric field distribution in XOY plane and XOZ plane of square microstructure absorber, circular microstructure absorber and annular microstructure absorber.

In order to investigate the superiority of the ring microstructure in more depth, we also studied the electric field distribution at 2803 nm (resonance absorption peak wavelength) for three models with square microstructure, circular microstructure and annular microstructure, as shown in Figure 6. As can be seen in Figure 6, the surfaces of the microstructures of all three geometries have a strong localized enhanced electric field, which means that SPRs resonance absorption appears on the surface of the microstructures [57]. However, the exciton resonance absorption of square microstructures mainly occur at the corners due to the higher electron density at the corners, which are more likely to interact with light waves. This situation is not ideal for the ability to limit light waves. While exciton resonance absorption occur at the surface for both circular and annular microstructures, the local field at the surface of the annular microstructure decays more slowly along the direction perpendicular to the surface, suggesting that the annular microstructure has a better confinement effect on the light waves. This is due to the fact that the annular microstructure has a smaller radial dimension, the exciton has a stronger vibrational intensity under the action of the light wave, and the interaction with the light wave is more inclined to the ideal resonance [58,59]. These are the reasons why the absorption performance of the ring microstructure is better than the other two microstructures.

**Figure 7:**
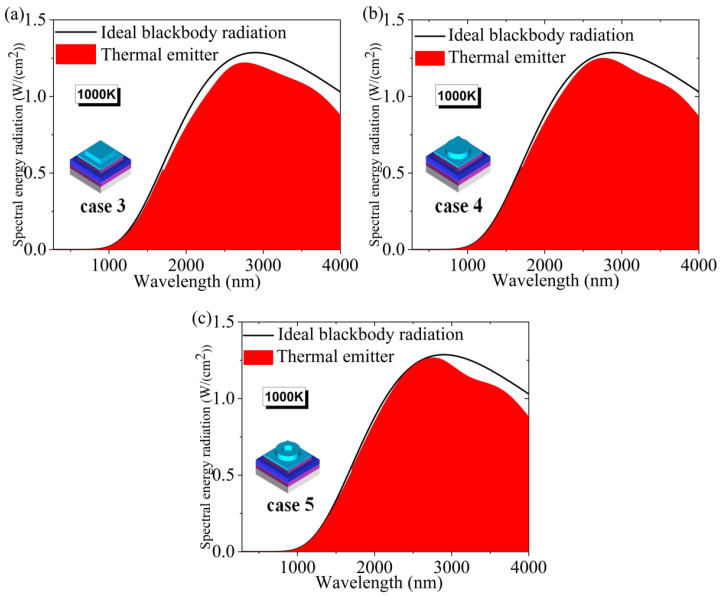
(**a**–**c**) Spectral radiation characteristics at 1000 K under three different microstructures.

Also, we calculated the thermal emission spectra of these three different microstructures at 1000 K, and the results are shown in Figure 7a–c. From the figure, we can see that the thermal emission performance of the model with the annular microstructure is also the best. In the 280–2750 nm band, the thermal radiation spectrum of the model with a annular microstructure is closer to that of an ideal blackbody. The thermal radiation efficiency at 1000 K was calculated to be 92.87% for the square microstructure, 94.95% for the circular microstructure, and 95.42% for the annular microstructure. It can be concluded that the model with a annular microstructure also has the highest thermal radiation efficiency. In conclusion, the annular microstructure is a superior model structure in terms of both absorption performance and thermal radiation performance [60,61].

**Figure 8:**
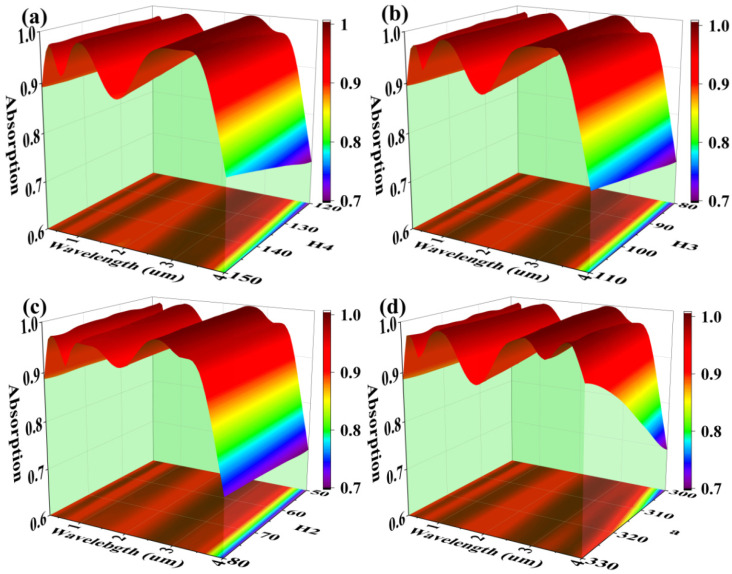
(**a**–**d**) are the comparison diagrams of absorption rates when H_4_, H_3_, H_2_ and a are changed.

The variation of structural parameters also has an effect on the absorption performance of the model. Therefore, we analyzed the changes in absorption properties by varying the thickness of the fourth Al_2_O_3_ film layer (H_4_), the thickness of the third Ti film layer (H_3_), the thickness of the second InAs film layer (H_2_) and the side length of the square microstructure (a). The results are shown in Figure 8a–d. It can be seen from Figure 8a that the absorption of the model in the 1500–2400 nm band diminishes with the increase of H_4_. This is due to the increased impedance of the metal-insulator layer caused by the increase in the thickness of the film layer, which results in a decrease in the absorption of SPRs at the metal-insulator layer interface [62,63]. This conclusion can also be drawn from the fact that the variation of the absorption properties in Figure 8b is almost identical to that in Figure 8a. The increase in the thickness of Ti metal layer (H_3_) also causes an increase in the impedance of the metal-insulator layer, which leads to a decrease in the absorption performance of the model [64,65]. From Figure 8c, it can be seen that the thickness (H_2_) variation of the second InAs film layer has a slight effect on the absorption performance of the model in the 1000–1600 nm band. This is due to the fact that the thickness of InAs nanosemiconductors determines the strength of the quantum-limited effect that appears. And the effect of the edge length of the microstructure (a) on the absorption performance is shown in Figure 8d. The increase in the edge length causes a decrease in the absorption performance of the model in the 1500–2300 nm band, but increases again in the 3700–4000 nm band. For the solar absorber model, the effect of this variation is not good. So the principle of choosing the edge length parameter is to keep it as small as possible [66]. However, too small will increase the difficulty of the process, so the structural parameters are chosen moderately to ensure the dual advantages of the model in theory and practice. The above is a discussion of the influence of structural parameters on the model.

**Figure 9:**
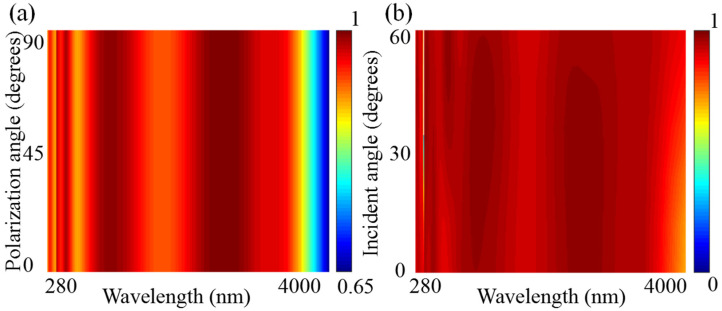
(**a**) Absorption diagram corresponding to different polarization angles. (**b**) Absorption diagram corresponding to different incident angles.

From a practical application point of view, the sunlight will certainly not be perfectly perpendicular to the absorber and completely free of polarization [67,68,69]. Therefore, we simulate the change of the absorption of the solar absorber when the polarization angle is from 0° to 90°. From Figure 9a we can conclude that the solar absorber is not affected by the polarization angle. This is because the solar absorber designed by us has full space symmetry, and the absorption rate corresponding to the incident light from TE to TM modes is the same [70,71,72]. At the same time, we also simulate the change of absorption when the angle of incident light changes from 0° to 60°. From Figure 9b we can conclude that changes in the angle of incidence have little effect on the absorption. Only in the very narrow wavelength range of about 395 nm, when the angle of incidence exceeds 15°, there is a large decrease in absorption, but this actually has little effect on the overall absorption. Even in the visible light band, when the incident angle is greater than 30°, the absorption increases. In the wavelength band of 3000–4000 nm, the absorption also increases with the increase of the incident angle, which is very beneficial to the absorption of the solar absorber.

## 4. Conclusions

In this paper, we propose an ultra-wideband high-efficiency solar absorber. After the simulation calculation of FDTD software, the absorption of the model in the 283–3615 nm band are over 90%, the absorption bandwidth is 3332 nm, and the average absorption is 95.66%. The weighted average absorption in the 280–4000 nm band was 95.78% under the light of AM1.5. At the same time, the reflectivity of the model in the 5586–20,000 nm band is all higher than 80%, with an average reflectivity of 94.52%. This indicates that the model has good spectral selectivity and excellent thermal infrared suppression. This advantage allows the model to work more stably under high temperature radiation conditions. By varying the structural parameters of the model one by one (under the condition of controlling other parameters to selected ideal values), the absorber was found to have a good process tolerance. Since the microstructure of the model is a highly symmetric annular structure, the model has polarization insensitivity. We also simulated the absorption performance of the model when the incident light is incident at 0–60°. The results show that the model has good wide-angle absorption properties. In addition, the model has a high thermal radiation efficiency of 95.42% in the 280–4000 nm band. The excellent thermal radiation performance makes the model have high potential for application in the field of thermophotovoltaic power generation.

## Figures and Tables

**Table 1:** A contrast with other solar absorbers.

References	Structure	Band-Width (>90%)	Absorption Efficiency	AM1.5 Absorption under Illumination
[43]	Ge cavity model	1868 nm	90% (250–3500 nm)	88% (250–4000 nm)
[44]	TiO_2_/TiN semi-circular grating model	475 nm	94% (380–760 nm)	Not studied
[45]	TiN nano disk model	1869 nm	93.77% (200–2600 nm)	95.89 (280–4000 nm)
[23]	GaAs grating composite structure based on W-Ti-GaAs	2350 nm	95% (500–2850 nm)	Not studied
this paper	6-layer structure of InAs ring microstructure model	3332 nm	95.66% (280–4000 nm)	95.78% (280–4000 nm)

## Data Availability

Publicly available datasets were analyzed in this study. This data can be found here: https://www.lumerical.com/ (accessed on 1 January 2020).
